# Predicting humoral responses to primary and booster SARS-CoV-2 mRNA vaccination in people living with HIV: a machine learning approach

**DOI:** 10.1186/s12967-024-05147-1

**Published:** 2024-05-07

**Authors:** Giorgio Montesi, Matteo Augello, Jacopo Polvere, Giulia Marchetti, Donata Medaglini, Annalisa Ciabattini

**Affiliations:** 1 Laboratory of Molecular Microbiology and Biotechnology, Department of Medical Biotechnologies, University of Siena, Siena, Italy; 2 Clinic of Infectious Diseases and Tropical Medicine, Department of Health Sciences, San Paolo Hospital, ASST Santi Paolo e Carlo, University of Milan, Milan, Italy

**Keywords:** Machine learning, SARS-CoV-2, HIV, Statistical modeling, Vaccines, mRNA, Antibodies, Immune response, ImmunoVirology

## Abstract

**Background:**

SARS-CoV-2 mRNA vaccines are highly immunogenic in people living with HIV (PLWH) on effective antiretroviral therapy (ART). However, whether viro-immunologic parameters or other factors affect immune responses to vaccination is debated. This study aimed to develop a machine learning-based model able to predict the humoral response to mRNA vaccines in PLWH and to assess the impact of demographic and clinical variables on antibody production over time.

**Methods:**

Different machine learning algorithms have been compared in the setting of a longitudinal observational study involving 497 PLWH, after primary and booster SARS-CoV-2 mRNA vaccination. Both Generalized Linear Models and non-linear Models (Tree Regression and Random Forest) were trained and tested.

**Results:**

Non-linear algorithms showed better ability to predict vaccine-elicited humoral responses. The best-performing Random Forest model identified a few variables as more influential, within 39 clinical, demographic, and immunological factors. In particular, previous SARS-CoV-2 infection, BMI, CD4 T-cell count and CD4/CD8 ratio were positively associated with the primary cycle immunogenicity, yet their predictive value diminished with the administration of booster doses.

**Conclusions:**

In the present work we have built a non-linear Random Forest model capable of accurately predicting humoral responses to SARS-CoV-2 mRNA vaccination, and identifying relevant factors that influence the vaccine response in PLWH. In clinical contexts, the application of this model provides promising opportunities for predicting individual vaccine responses, thus facilitating the development of vaccination strategies tailored for PLWH.

**Supplementary Information:**

The online version contains supplementary material available at 10.1186/s12967-024-05147-1.

## Background

Since the beginning of the SARS-CoV-2 pandemic, people living with HIV (PLWH) have been considered at higher risk of serious illness and severe outcomes from COVID-19. Despite conflicting data emerged from preliminary analyses conducted in small cohorts [1, 2], subsequent larger observational studies confirmed that PLWH may suffer worse COVID-19 outcomes compared to the general population, especially in the presence of scarce immune reconstitution despite antiretroviral therapy (ART) and in case of unsuppressed HIV replication [3–8].

Owing to such vulnerability, PLWH were prioritized for SARS-CoV-2 vaccine administration since the early phases of the vaccination campaign. Research conducted to date agrees that, overall, PLWH mount immune responses to the primary cycle of SARS-CoV-2 vaccine which are comparable to those developed by HIV-negative people [3, 9]. However, when assessing HIV-specific factors typically related to adverse outcomes, such as low CD4 T-cell counts, inverted CD4/CD8 ratio, and uncontrolled HIV viremia, they invariably appeared associated to impaired cellular and humoral responses [3, 9–12], suggesting that PLWH with poor immune restoration and/or ongoing HIV replication should receive booster doses. An additional vaccine dose has been shown to substantially improve humoral responses in PLWH with hyporesponse after primary cycle [13–15]. However, whether HIV-related viro-immunological parameters or other factors may have an impact on immune responses to booster vaccination in PLWH is unclear [13, 16–18], yet it would be of utmost importance to personalize boosting strategies in the current phase of shifting from the pandemic to the endemic stage of COVID-19.

Generally, in biological contexts where regression analysis is required to study associations between variables, linear regression models alongside various feature selection strategies are commonly used [19, 20]. In recent years, advancements in machine learning strategies have enabled the quantification of both linear and non-linear associations in an unbiased manner and provided a comprehensive characterization of more intricate and complex interactions among predictor variables of a certain outcome [21]. Such approaches have been employed to identify key clinical factors associated with antibody responses and to predict vaccine immunogenicity in fragile and immunosuppressed populations such as organ transplant recipients [22, 23]. However, the utility of these algorithms in predicting immune responses to SARS-CoV-2 vaccines in PLWH has not been fully explored.

In the present study, we compared different machine learning algorithms in the setting of a large observational study involving 497 PLWH after primary and booster SARS-CoV-2 mRNA vaccination, to develop a model able to accurately predict vaccine-elicited humoral immunity and identify relevant factors that influence vaccine response over time in this vulnerable population.

## Methods

### Study design

The San Paolo Infectious Diseases HIV-Vax (SPID-HIV-Vax) is a prospective observational study which was established in March 2021 that enrolled 800 PLWH who received the anti-SARS-CoV-2 Spikevax^™^ mRNA vaccine (Moderna) at the Clinic of Infectious Diseases and Tropical Medicine, San Paolo Hospital, ASST Santi Paolo e Carlo, Department of Health Sciences, University of Milan, Milan, Italy. Data concerning demographic characteristics, comorbidities, HIV-related features and self-reported previous SARS-CoV-2 infection were collected at enrollment using RedCap electronic data capture tools [24]. PLWH within SPID-HIV-Vax cohort were eligible to participate in the present study if they met the following criteria: availability of at least one post-vaccine anti-S IgG determination between March 2021 and January 2023, and availability of all baseline demographic and clinical variables included in the original database.

The study was approved by the local Ethical Committee and written informed consent was obtained from each participant.

### Biological samples collection and antibody quantification

**Fig. 1:**
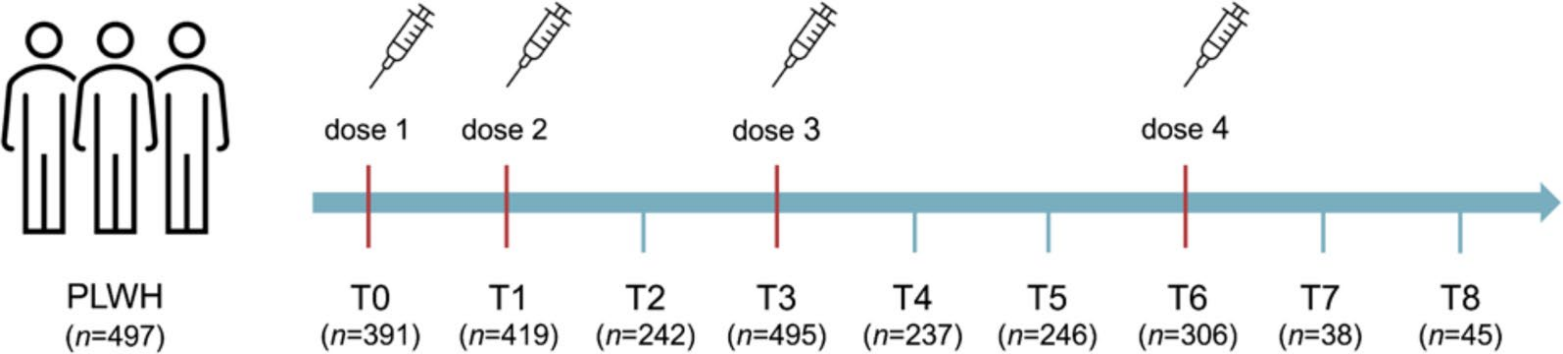
Clinical study design. Blood samples were collected at each longitudinal time-point (T) from PLWH receiving multiple doses of mRNA SARS-CoV-2 vaccines. Number of available anti-S IgG samples are reported for each time-point. PLWH: people living with HIV; T0: day of the first dose; T1: 1 month after the first dose, coinciding with the day of the second dose; T2: 1 month after the second dose; T3: 6 months after the second dose, coinciding with the third dose administration; T4: 1 month after the third dose; T5: 6 months after the third dose; T6: 12 months after the third dose, coinciding with the fourth dose administration; T7: 1 month after the fourth dose; T8: 6 months after the fourth dose

From each participant, venous peripheral blood samples were collected at the following time points: day of first dose (T0); 1 month after first dose—coinciding with the day of second dose—(T1); 1 month after second dose (T2); 6 months after second dose—coinciding with third dose administration—(T3); 1 month after third dose (T4); 6 months after third dose (T5); 12 months after third dose—coinciding with fourth dose administration—(T6); 1 month after fourth dose (T7); 6 months after fourth dose (T8) (Fig. 1). Anti-trimeric Spike (S) IgG antibodies were quantitatively determined in serum samples by the LIAISON® SARS-CoV-2 TrimericS IgG assay (DiaSorin, Italy), and concentration expressed as binding antibody units per milliliter (BAU/mL).

### Statistical and machine learning models

To build a model capable of predicting antibody response to vaccination based on available demographic and clinical parameters both linear and non-linear regression methods were employed and compared. Variables were normalized using z-score transformation when numeric and converted into dummy variables when categorical. The independent variables used as predictors encompassed demographic characteristics, comorbidities, viro-immunological HIV-related parameters, HIV epidemiology, and ART, totaling 39 variables. A temporal variable accounting for the days elapsed since the first vaccine dose administration was also included to build all algorithms. All models employed a train-and-test strategy, with the 80% of the dataset used as the training set, while the remaining 20% as the test set for evaluating model-performances. The metrics used to assess model quality were calculated as the mean values obtained through a fivefold cross-validation (CV): R-squared (R^2^) and Root Mean Squared Error (RMSE) for both linear and non-linear models. Only for linear models, Akaike Information Criterion (AIC) and Bayesian Information Criterion (BIC) were computed during the training phase.

#### Generalized linear models

For the construction of linear models, the *glm* function from the stats package v3.6.2 via R statistical software v4.3.1 was employed. Feature selection was performed by Stepwise and Multi-Model inference to mitigate high dimensionality and multicollinearity issues.

Stepwise variable selection was executed employing both forward (SF) and backward (SB) approaches by means of the pre-built-in *stepAIC* function in R. Multi-model (MM) inference was conducted using the *glmulti* package v1.0.8 by generating all possible linear combinations of the independent variables and testing each model with the aim of AIC minimization. Due to the impracticality of testing all possible linear combinations with all the variables, dummy variables related to comorbidities (12 variables) were excluded. Thus, over 4 million combinations were tested, with each iteration fitting a *glm* via an exhaustive screening method.

#### Non-linear models

Tree Regression and Random Forest analyses were conducted using the *rpart* v4.1.23 and the *randomForest* packages v4.7–1.1 in R, respectively. Regarding Tree Regression, an analysis of variance (ANOVA) was chosen as the criterion for assessing node split quality, while default settings were retained for all other parameters. For Random Forest, hyperparameter tuning led to the selection of 300 trees as the optimal parameter within the range of 10–10,000. Variable importances were computed using two measures: *%IncMSE*, assessing the rise in MSE resulting from the removal of the variable; *IncNodePurity*, quantifying the increase in residual sum of squares attributable to the exclusion of the variable.

### Statistical analysis

Continuous variables were expressed as median (interquartile range, IQR), and categorical variables as number, *n* (percentage, %). Correlation analyses employed the Spearman correlation coefficient, by the *ggpubr* package v0.6.0 in R. A *p*-value ≤ 0.05 was considered significant. Visualizations were generated using *ggplot2* and *plotmo* libraries in R v4.3.

## Results

### Study population

A total of 497 PLWH within the SPID-HIV-Vax cohort met the inclusion criteria for the present study. The vaccination schedule and blood sample collection are reported in Fig. 1. Baseline characteristics of the study participants are reported in detail in Table 1. Briefly, median age was 54 (IQR: 44–59) years, and 408 (82.1%) were males. Median time from HIV diagnosis was 12 (IQR: 7–22) years. Median CD4 T-cell *nadir* was 220 (IQR: 81–370) cells/µL; current CD4 T-cell count was 701 (IQR: 512–934) cells/µL with a median CD4/CD8 ratio of 0.81 (IQR: 0.56–1.14). All participants have been on ART for a median of 9 (IQR: 5–15) years, and 483 (97.18%) had undetectable plasma HIV-RNA. Thirty-seven (7.44%) PLWH reported SARS-CoV-2 infection prior to vaccination.

**Table 1:** Demographic and HIV-related characteristics of PLWH at baseline

Characteristics	PLWH (*n* = 497)
Age, years, median (IQR)	54 (44–59)
Sex at birth, *n* (%)	
Male	408 (82.1)
Female	89 (17.9)
Ethnicity, *n* (%)	
Caucasian	428 (86.12)
Arab	9 (1.81)
Latin	41 (8.25)
African	6 (1.21)
Asian	13 (2.61)
Epidemiology, *n* (%)	
MSM	254 (51.11)
MSW/WSM	148 (29.79)
Transgender	1 (0.2)
IDU	71 (14.29)
Vertical transmission	5 (1.01)
Other/unknown	18 (3.6)
Comorbidities, *n* (%)	
None	279 (56.14)
Hypertension	101 (20.32)
Cardiovascular disease	52 (10.46)
Cerebrovascular disease	20 (4.02)
Chronic kidney disease	30 (6.05)
COPD/Asthma	33 (6.64)
Peptic ulcer	12 (2.41)
Hemiplegy	9 (1.81)
Previous leukemia/lymphoma	19 (3.82)
Previous solid cancer	35 (3.82)
Metastatic solid cancer	9 (1.81)
Autoimmune diseases	48 (9.66)
Neurologic disease	30 (6.05)
Charlson Comorbidity Index, median (IQR)	0 (0–1)
BMI, median (IQR)	24.62 (22.66–27.46)
BMI strata, *n* (%)	
< 18.5	19 (3.8)
18.5–24.99	249 (50.1)
25–29.9	174 (35)
≥ 30	55 (11.1)
Viro-immunologic parameters, median (IQR)	
CD4 T-cell *nadir*, cells/µL	220 (81–370)
CD4 T-cell count, cells/µL	701 (512–934)
CD4 T-cell percentage	32 (25–39)
CD8 T-cell count, cells/µL	885 (640–1156)
CD8 T-cell percentage	40 (33–47)
CD4/CD8 ratio	0.81 (0.56–1.14)
CD4 T-cell count strata, *n* (%)	
< 200 cells/µL	20 (4.02)
200–500 cells/µL	95 (19.12)
> 500 cells/µL	382 (76.86)
CD4/CD8 ratio ≥ 1, *n* (%)	174 (18.91)
Undetectable HIV-RNA (< 50 copies/mL), *n* (%)	483 (97.18)
Previous AIDS diagnosis, *n* (%)	150 (30.18)
Time from HIV diagnosis, years, median (IQR)	12 (7–22)
Current ART regimen, *n* (%)	
INSTI-based	361 (72.64)
PI-based	34 (6.84)
INSTI + PI-based	8 (1.61)
NNRTI-based	94 (18.91)
Duration of ART, years, median (IQR)	9 (5–15)
Previous SARS-CoV-2 infection, *n* (%)	37 (7.44)
Time between prime and booster vaccination, days, median (IQR)	176 (162–190)

*IQR* interquartile range, *MSM* men who have sex with men, *MSW* men who have sex with women, *WSM* women who have sex with men, *IDU* injective drug use, *COPD* chronic obstructive pulmonary disease, *BMI* body mass index, *AIDS* acquired immunodeficiency syndrome, *INSTI* integrase strand-transfer inhibitor, *PI* protease inhibitor, *NNRTI* non-nucleoside reverse transcriptase inhibitor

### Regression models selection

To develop a predictive model for the antibody concentrations at each sequential time point following the administration of SARS-CoV-2 vaccine, linear and non-linear models were constructed as described above.

#### Generalized linear models

All three GLM models—derived from SF, SB and MM feature selection approaches—identified a positive relationship of antibody concentrations with the presence of a prior SARS-CoV-2 infection, previous AIDS events, and duration of ART, whilst a negative association with CD8 T-cell percentage (Additional file 1: Tables S1–S3). Additionally, SF and SB models revealed a positive dependence of vaccine-elicited anti-S IgG on sex at birth (female) and heterosexual behavior, while a negative correlation with Caucasian ethnicity. Specifically, SF model also identified a negative association with time between primary and booster vaccination, as well as with HIV-RNA copies in plasma, while SB model found a significant positive dependence on BMI. Lastly, the MM inference approach uncovered a positive relationship with BMI and a negative one with CD4 T-cell *nadir*. Overall, these three linear models showed a generally low cross-validated R^2^ and elevated cross-validated RMSE indicative of suboptimal performances on the test dataset (Table 2). Consequently, a shift towards non-linear methodologies was employed to capture the intricate relationships among variables more accurately.

**Table 2:** Linear and non-linear model performances

Name	Model	AIC	BIC	CV R^2^	CV RMSE
Stepwise-forward	*glm*	4047.2	4123.2	0.357	0.801
Stepwise-backward	*glm*	4072.1	4169.8	0.358	0.801
glmulti	*glm*	4079.9	4134.2	0.351	0.804
Tree regression	*rpart*	–	–	0.795	0.451
Random forest	*randomForest*	–	–	0.845	0.412

R^2^ (CV-R^2^) and Root Mean Squared Error (CV-RMSE) metrics have been computed on test set using a fivefold cross-validation approach

*AIC* Akaike Information Criterion obtained on the train set, *BIC* Bayesian Information Criterion computed on the train set

#### Non-linear models

The Tree Regression model exhibited enhanced performances compared to its linear counterparts (CV-R^2^ = 0.795, CV-RMSE = 0.451) (Table 2). The optimal tree configuration identified 12 variables, including previous SARS-CoV-2 infection, BMI, plasma HIV-RNA and HIV-related viro-immunological parameters (Additional file 1: Fig S1). Random Forest outperformed the preceding methodologies in terms of both highest R^2^ and lowest RMSE (CV-R^2^ = 0.845, CV-RMSE = 0.412) (Table 2), leading to its selection as the optimal model for subsequent analysis.

### Antibody prediction through random forest regression

**Fig. 2:**
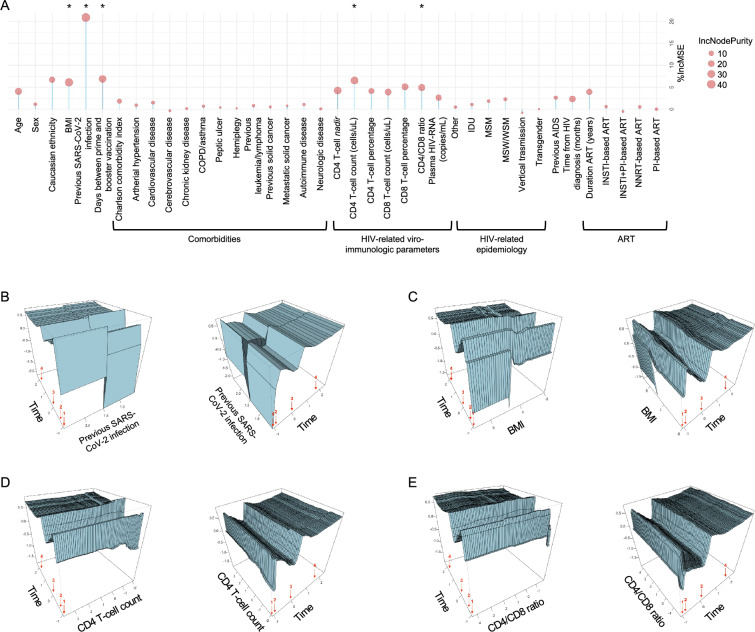
Random Forest regression analysis. **A** Variable importance resulting from Random Forest model. Variable importances are expressed in terms of the rise in MSE resulting from the removal of the variable (namely %IncMSE, y-axis) and the increase in residual sum of squares attributable to the exclusion of the variable (namely IncNodePurity, sphere dimension). Top 5 selected important variables are marked with an asterisk (*). **B**–**E** Interaction between top important variables and time since first vaccine dose within the Random Forest model. 3D partial dependence plots are generated by plotting the predicted response [anti-S IgG expressed in binding antibody units per milliliter (BAU/mL)] on the z-axis as two variables are changed (all other variables held at their median/mode values). Numerical variables are normalized using z-score. Red arrows indicate the number of vaccine doses received. For each pair of parameters two different visualizations were obtained by rotating the plot around the z-axis with an angle of 80°. The interaction of time since first dose administration, reported on the x-axis, versus previous SARS-CoV-2 infection (**B**), BMI (**C**), CD4 T-cell count (**D**), and CD4/CD8 ratio (**E**). *BMI* body mass index, *COPD* chronic obstructive pulmonary disease, *IDU* injective drug use, *MSM* men who have sex with men, *MSW* men who have sex with women, *WSM* women who have sex with men, *AIDS* acquired immunodeficiency syndrome, *ART* antiretroviral therapy, *INSTI* integrase strand-transfer inhibitor, *PI* protease inhibitor, *NNRTI* non-nucleoside reverse transcriptase inhibitor

The selected Random Forest regression approach identified, among 39 different clinical, demographic, and immunological factors, several variables as more influential in predicting the antibody response, such as demographic variables (age and Caucasian ethnicity), BMI, previous SARS-CoV-2 infection, the number of days between the second and third vaccine doses, HIV-related viro-immunological parameters, time since HIV infection acquisition, and duration of ART (Fig. 2A). The top 5 variables, in terms of both %*IncMSE* and *IncNodePurity*, in order of importance, were previous SARS-CoV-2 infection, BMI, CD4 T-cell count, days between prime and booster vaccination, and CD4/CD8 ratio. To investigate the role of these variables in predicting the antibody response over time, the variable “days between prime and booster vaccination” was excluded since it could not impact time points preceding the third dose. The temporal influence of each of the selected 4 variables on the prediction of the antibody response within the Random Forest model was investigated using 3D partial dependence plots. The graphs were obtained by pairing each variable of interest with the temporal one, while setting all other variables (background variables) to their median value (for continuous variables) or mode (for categorical variables) (Fig. 2B–E).

A pronounced dependence of the predicted antibody response was observed at earlier time points after the administration of the primary vaccine cycle for all selected variables. This dependence gradually diminished over time with the consequent administration of vaccine doses, culminating in a uniform humoral response for each value assumed by the examined variable. Specifically, a positive relationship was observed with the presence of a previous SARS-CoV-2 infection before vaccine administration (Fig. 2B), while reduced antibody concentrations were noted for lower values of BMI, CD4 T-cell count, and CD4/CD8 ratio (Fig. 2C–E).

In quantitative terms, the declining impact over time of the top 5 variables on the antibody response was corroborated by a Spearman correlation analysis conducted across the entire study population at each time point (Additional file 1: Fig S2). Notably, similar trends to those highlighted by applying the Random Forest model were observed, since the significance of the correlation showed a diminishing trend with the increasing number of vaccine doses received over time. This alignment between the correlation analysis and the Random Forest model outcomes reinforces the temporal evolution of variables’ influence on the antibody response, emphasizing the consistency of observations across analytical methodologies.

## Discussion

In this study, we leveraged data from a longitudinal observational study to train and test different machine learning algorithms to develop a predictive model of immune responses to SARS-CoV-2 mRNA primary and booster vaccination in PLWH. The specific aims were to forecast vaccine-elicited humoral responses in this vulnerable population based on several demographic and clinical information that may be easily retrieved from electronic charts in clinical practice settings, and to simultaneously analyze the impact of these variables on antibody production over time.

We found that, while commonly used linear regression models show suboptimal performances, non-linear methodologies display a significantly better ability to capture the intricate relationships among variables. In particular, Random Forest regression resulted as the best performing algorithm in predicting vaccine-induced antibody response. This is likely attributable to the fact that the various feature selection strategies employed in linear models often lead to the exclusion of important variables that, when considered individually, may have a minor role, whereas in a multi-variable context they would assume a stronger predictive role [25, 26].

Notably, the key clinical factors influencing the vaccine humoral immunogenicity that were identified by the Random Forest model were: previous SARS-CoV-2 infection, CD4 T-cell count, CD4/CD8 ratio, BMI, and time between primary vaccination cycle and booster dose. In detail, SARS-CoV-2 infection before vaccine administration appeared to positively influence the vaccine-elicited antibody levels. By contrast, low CD4 T-cell counts, CD4/CD8 ratio and BMI values were associated with reduced antibody responses to the vaccine. Lastly, increasing time between primary vaccination cycle and booster dose was associated with higher antibody levels after the administration of booster doses. Remarkably, these key clinical factors, identified through the Random Forest machine learning approach, are congruent with clinical and laboratory observations [3, 9, 10, 12, 27, 28]. This corroborates and supports the validity of our model, confirming it as an important tool for future prediction studies. Indeed, hybrid immunity, derived from a combination of both natural infection and vaccination, has been shown to ensure immune protection which is higher in both magnitude and durability than that provided by either vaccine or infection alone [29]. Furthermore, poor immune recovery despite ART has been distinctly associated with reduced humoral and T-cell responses to SARS-CoV-2 vaccines in PLWH [3, 9–12]. Similarly to obese people, underweight ones develop weaker immune responses to SARS-CoV-2 vaccines [28], due to a severe impairment of the immune system [30]. Lastly, several studies demonstrated that an extended interval between SARS-CoV-2 mRNA vaccine doses results in stronger humoral responses [31–34], owing to a decline in antibody levels which limits the Fc-mediated clearance of vaccine-encoded antigens, thus allowing de novo priming of B cells [34].

In this context, our machine learning approach expands the knowledge of the modeling strategies to be employed in studies aiming to predict outcomes involving complex biological mechanisms. Indeed, we demonstrated that such associations are not linear and thus more nuanced than previously believed, due to the reciprocal interactions between such factors in influencing vaccine-induced humoral responses.

Additionally, while previous research mainly focused on the primary vaccine cycle, the present study extends the knowledge on immune responses to booster doses in this vulnerable population. In this respect, a reduction of the model dependence from the identified predictors over time was observed, revealing that while the aforementioned factors may play a critical role in dictating humoral immunogenicity to the primary vaccine cycle in PLWH, the importance of their role significantly wane over time, so that antibody responses to booster shots are uniform across the entire population regardless demographic and clinical features.

Some limitations need to be acknowledged in this study. Firstly, the model herein presented was developed using data derived from individuals vaccinated with the Spikevax™ mRNA vaccine (Moderna), and thus may not directly translate to PLWH receiving other SARS-CoV-2 vaccines or heterologous vaccine combinations. Additionally, while previous SARS-CoV-2 infection was recorded at baseline, data on breakthrough infections during the follow-up period were not available. Lastly, the sample size was relatively small, especially for latest time points. Expanding the scope of the investigation to encompass different vaccine platforms and heterologous prime-boost combinations, alongside with data from other cohorts of PLWH and other fragile populations and vaccine antigens, will strengthen such findings, providing valuable insights for the design of future vaccination strategies.

## Conclusions

This study showcases that machine learning algorithms capable of quantifying non-linear associations allow to accurately predict humoral responses to SARS-CoV-2 mRNA primary and booster vaccination in PLWH by employing clinical, demographic and HIV-related variables commonly available in medical charts. While low CD4 T-cell counts, CD4/CD8 ratio and BMI are associated with poor immunogenicity of the primary vaccine cycle, the administration of additional doses overcome the negative influence of these factors, suggesting that further booster doses should be offered to PLWH. Moreover, the application of this model in clinical contexts holds potential promise for public health strategies, empowering clinicians to predict individual humoral responses to vaccination, simply using demographic and clinical information that may be easily retrieved in medical practice settings. As a result, our model not only contributes to a deeper understanding of vaccine responsiveness but also offers practical guidance for implementing effective and targeted vaccination strategies in PLWH that can be particularly helpful for improving possible epidemic or pandemic vaccination policies.

### Supplementary Information

**Additional file 1: Table S1.** Summary of Forward model regression analysis. **Table S2.** Summary of Backward model regression analysis. **Table S3.** Summary of Multi-Model regression analysis. **Figure S1.** Tree Regression model importance plot. Variables selected as most important and used to build the final optimal tree of the model. Variable importances, calculated by rpart package, are reported on both x-axis and sphere radious. ART: antiretroviral therapy; BMI: body mass index. **Figure S2.** Spearman correlation analysis. Correlation analysis performed between the top 5 important variables selected from the Random Forest model (y-axis) and anti-S IgG (binding antibody units per milliliter (BAU/mL)) at each time point (x-axis). R^2^ values are expressed as colour gradient ranging from violet to yellow. Numerical P-values of each pairwise comparison are reported within each box. BMI: body mass index; NA: not applicable; T1: 1 month after the first dose, coinciding with the day of the second dose; T2: 1 month after the second dose; T3: 6 months after the second dose, coinciding with the third dose administration; T4: 1 month after the third dose; T5: 6 months after the third dose; T6: 12 months after the third dose, coinciding with the fourth dose administration; T7: 1 month after the fourth dose; T8: 6 months after the fourth dose.

## Data Availability

The datasets generated during the current study are not publicly available because they contain sensitive data to be treated under data protection laws and regulations. Appropriate forms of data sharing can be arranged after a reasonable request to the corresponding author.
