# Supplementary material for: Predicting humoral responses to primary and booster SARS-CoV-2 mRNA vaccination in people living with HIV: a machine learning approach
Source: J Transl Med. 2024 May 7;22:432. doi: 10.1186/s12967-024-05147-1 (PMC11077794; doi:10.1186/s12967-024-05147-1)
Supplement: Supplementary file 1 — Additional file 1: Table S1. Summary of Forward model regression analysis. Table S2. Summary of Backward model regression analysis. Table S3. Summary of Multi-Model regression analysis. Figure S1. Tree Regression model importance plot. Variables selected as most important and used to build the final optimal tree of the model. Variable importances, calculated by rpart package, are reported on both x-axis and sphere radious. ART: antiretroviral therapy; BMI: body mass index. Figure S2. Spearman correlation analysis. Correlation analysis performed between the top 5 important variables selected from the Random Forest model (y-axis) and anti-S IgG (binding antibody units per milliliter (BAU/mL)) at each time point (x-axis). R2 values are expressed as colour gradient ranging from violet to yellow. Numerical P-values of each pairwise comparison are reported within each box. BMI: body mass index; NA: not applicable; T1: 1 month after the first dose, coinciding with the day of the second dose; T2: 1 month after the second dose; T3: 6 months after the second dose, coinciding with the third dose administration; T4: 1 month after the third dose; T5: 6 months after the third dose; T6: 12 months after the third dose, coinciding with the fourth dose administration; T7: 1 month after the fourth dose; T8: 6 months after the fourth dose. [file 12967_2024_5147_MOESM1_ESM.pptx]

## Slide 1
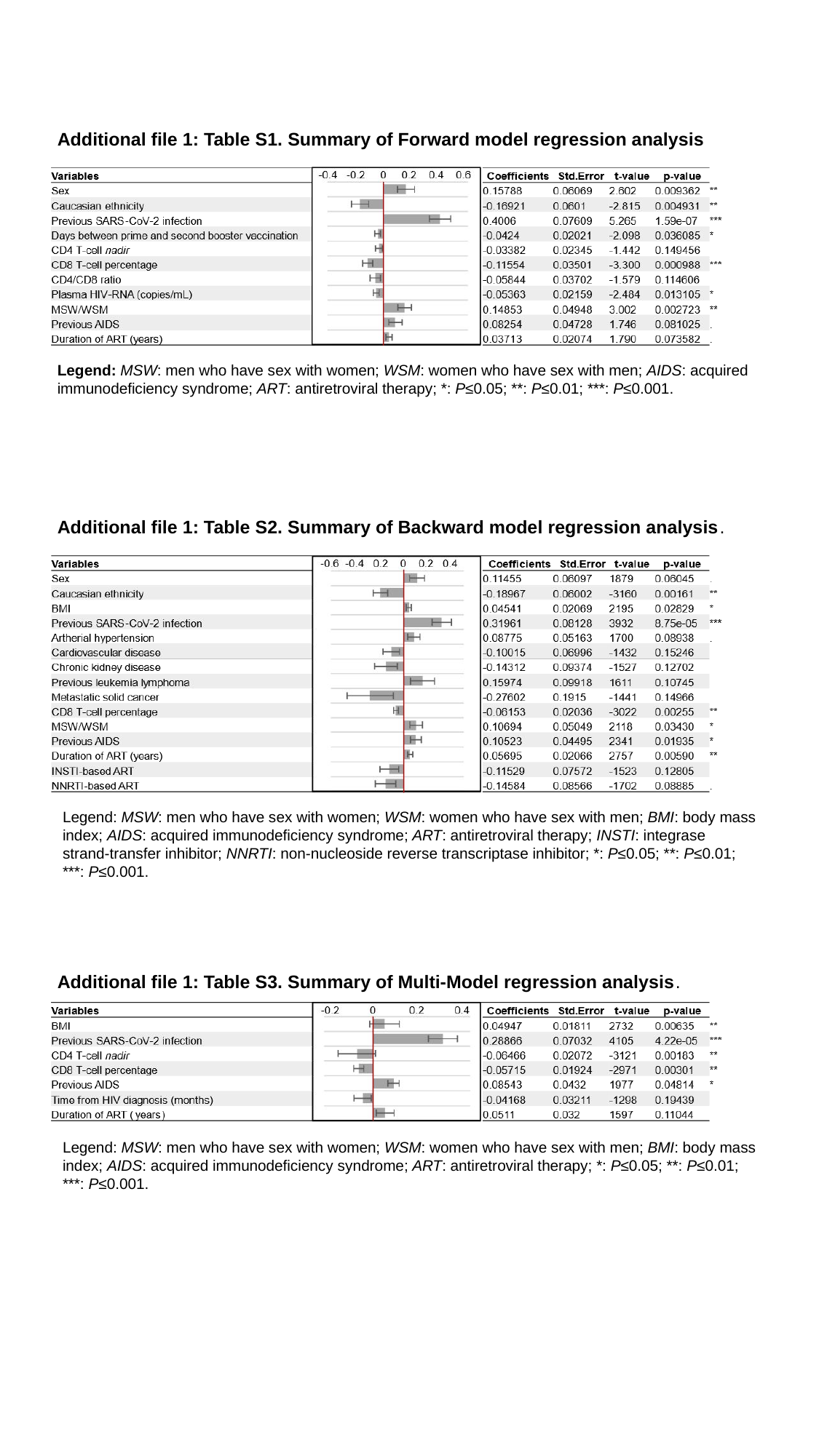

Additional file 1: Table S1. Summary of Forward model regression analysis
Legend: MSW: men who have sex with women; WSM: women who have sex with men; AIDS: acquired immunodeficiency syndrome; ART: antiretroviral therapy; *: P≤0.05; **: P≤0.01; ***: P≤0.001.
Additional file 1: Table S2. Summary of Backward model regression analysis.
Legend: MSW: men who have sex with women; WSM: women who have sex with men; BMI: body mass index; AIDS: acquired immunodeficiency syndrome; ART: antiretroviral therapy; INSTI: integrase strand-transfer inhibitor; NNRTI: non-nucleoside reverse transcriptase inhibitor; *: P≤0.05; **: P≤0.01; ***: P≤0.001.
Additional file 1: Table S3. Summary of Multi-Model regression analysis.
Legend: MSW: men who have sex with women; WSM: women who have sex with men; BMI: body mass index; AIDS: acquired immunodeficiency syndrome; ART: antiretroviral therapy; *: P≤0.05; **: P≤0.01; ***: P≤0.001.

## Slide 2
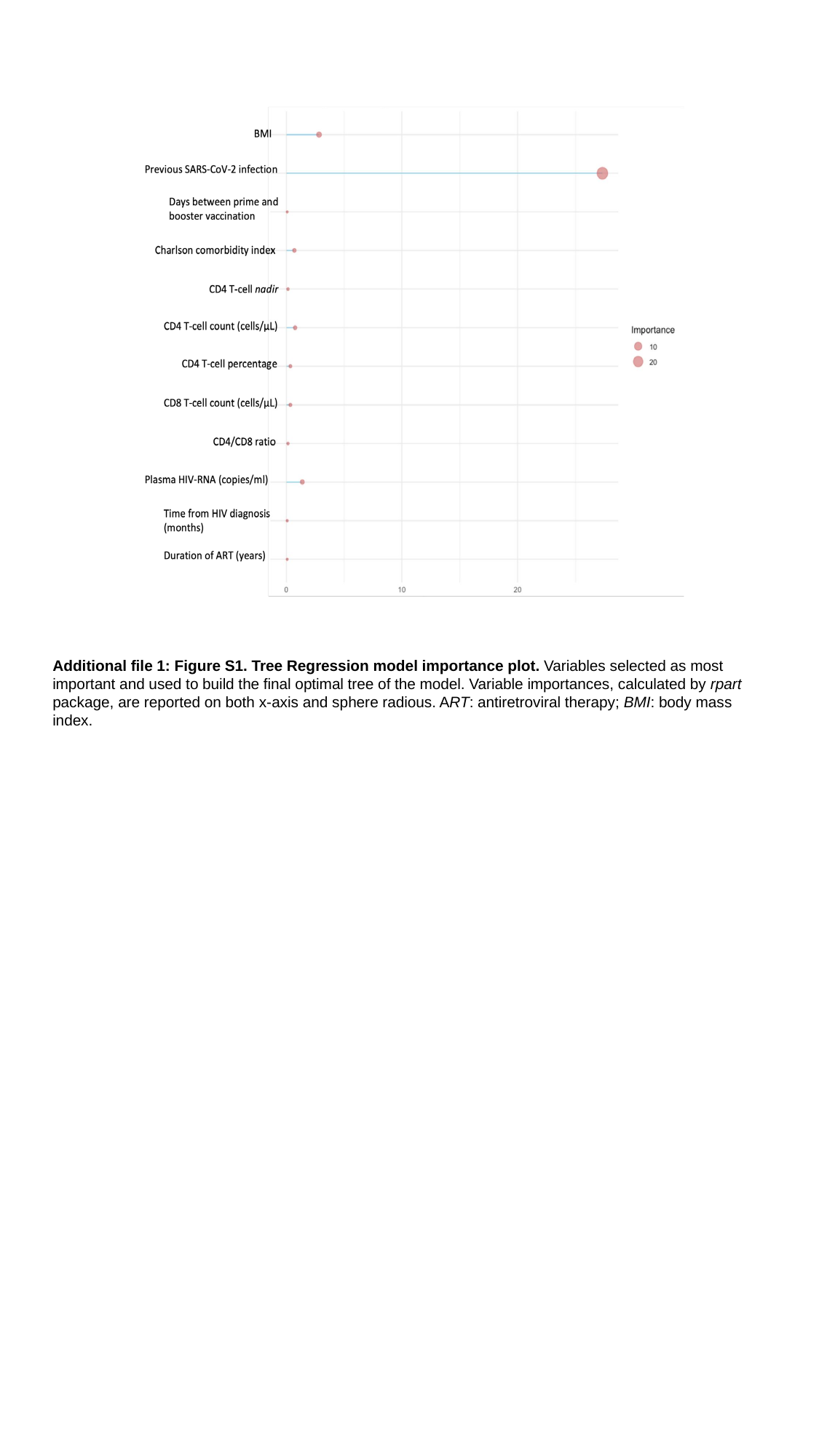

Additional file 1: Figure S1. Tree Regression model importance plot. Variables selected as most important and used to build the final optimal tree of the model. Variable importances, calculated by rpart package, are reported on both x-axis and sphere radious. ART: antiretroviral therapy; BMI: body mass index.

## Slide 3
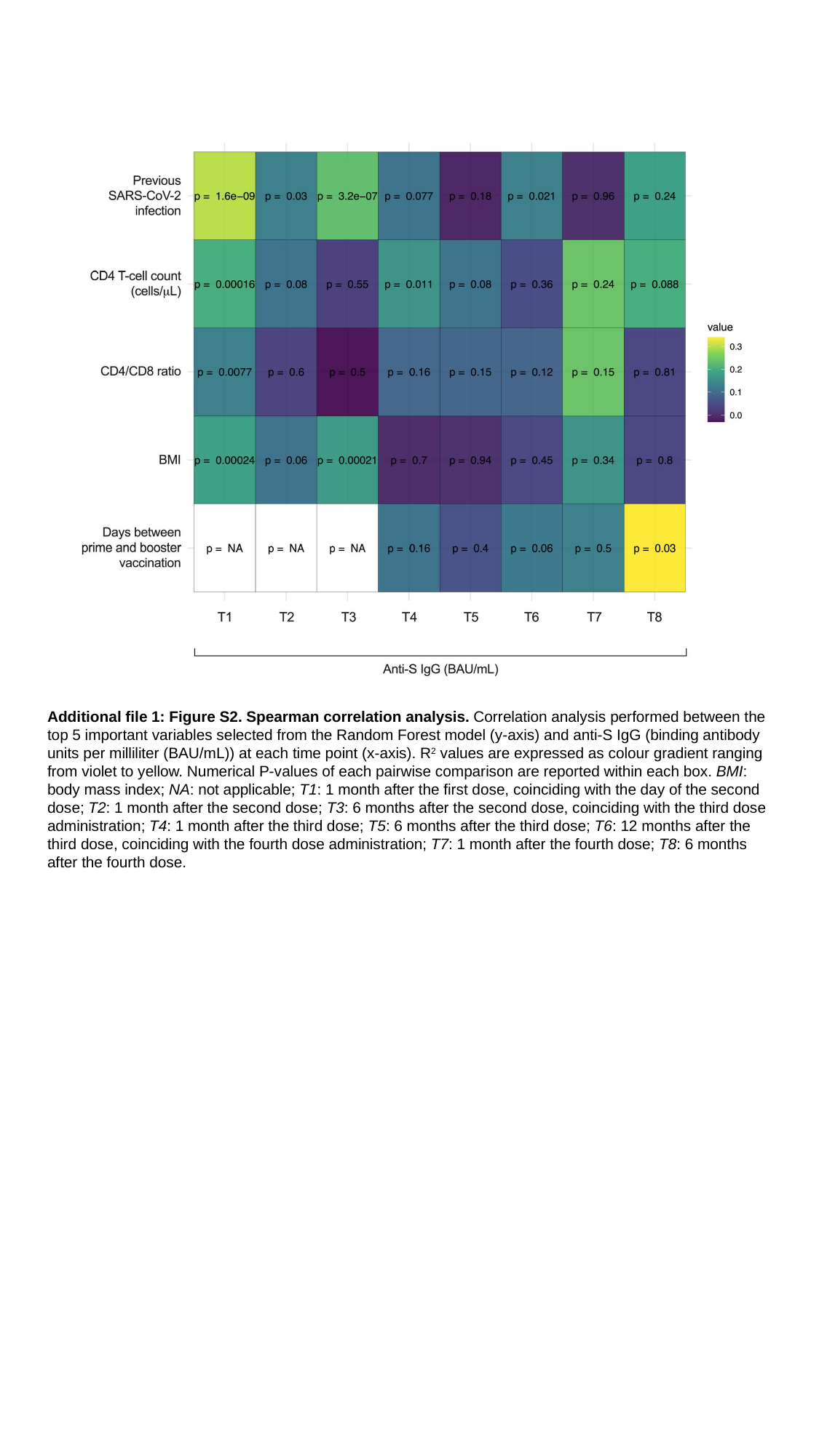

Additional file 1: Figure S2. Spearman correlation analysis. Correlation analysis performed between the top 5 important variables selected from the Random Forest model (y-axis) and anti-S IgG (binding antibody units per milliliter (BAU/mL)) at each time point (x-axis). R2 values are expressed as colour gradient ranging from violet to yellow. Numerical P-values of each pairwise comparison are reported within each box. BMI: body mass index; NA: not applicable; T1: 1 month after the first dose, coinciding with the day of the second dose; T2: 1 month after the second dose; T3: 6 months after the second dose, coinciding with the third dose administration; T4: 1 month after the third dose; T5: 6 months after the third dose; T6: 12 months after the third dose, coinciding with the fourth dose administration; T7: 1 month after the fourth dose; T8: 6 months after the fourth dose.
